# Deciphering the bone marrow microenvironment’s role in multiple myeloma immunotherapy resistance

**DOI:** 10.3389/fimmu.2025.1613265

**Published:** 2025-07-18

**Authors:** Nicolas Thomas Iannozzi, Nicola Giuliani, Paola Storti

**Affiliations:** ^1^ Department of Medicine and Surgery, University of Parma, Parma, Italy; ^2^ Hematology, Azienda Ospedaliero-Universitaria di Parma, Parma, Italy

**Keywords:** multiple myeloma, resistance, mesenchymal cells, car-t, bi-specific antibodies

## Abstract

Multiple Myeloma (MM) is a malignant monoclonal gammopathy characterized by the proliferation of plasma cells (PC) in the bone marrow (BM). The tight cross-talk between the BM microenvironment and PC is the hallmark of MM. The BM microenvironment comprises a cellular compartment, consisting of hematopoietic and non-hematopoietic cells. The first includes myeloid cells, T- and B-lymphocytes, natural killer (NK) cells, macrophages, and osteoclasts (OCs). In contrast, non-hematopoietic cell types include BM-derived mesenchymal stromal cells (MSCs), osteoblasts, adipocytes and endothelial cells. Besides the cellular compartment, there is a non-cellular compartment that includes extracellular matrix, growth factors, chemokines, and several cytokines. All these members play distinctive but interacting roles in the progression of MM and the drug response. MM remains an incurable disease, but in the last years immunotherapy has emerged as an important tool in the treatment of MM. The involvement of the BM microenvironment is a relevant barrier in the response to immunotherapy and in generating resistance. In this review, we provide an overview of the BM microenvironment perturbation in MM patients and how it can determine the possible resistance to immunotherapy, including monoclonal antibodies (mAbs), antibody-drug conjugates, chimeric antigen receptor T-cell (CAR-T), and bispecific T-cell engagers (BsAbs).

## 1 Introduction

Multiple myeloma (MM) is a plasma cell (PC) malignancy that develops into the bone marrow (BM), where it establishes close interaction with surrounding cells, resulting in tumor growth, survival, and drug resistance. BM microenvironment can support the expansion of MM PCs, evasion of immune surveillance by inducing abnormalities in immune cells [natural killer cells (NK), dendritic cells (DC), and T-cells] and by enhancing the release of immunoregulatory cytokines (1, 2).

Over the past decades, a deeper understanding of the complex MM pathophysiology has prompted drug development and clinical practice, resulting in significant improvements in patient outcome. The standard therapy to treat newly diagnosed MM (NDMM) patients is induction therapy based on quadruplets drug combination including anti-CD38 monoclonal antibody (mAb) followed by high-dose chemotherapy plus autologous stem cell transplant (ASCT) for young patients (3). On the other hand, patients not eligible for transplantation mainly receive treatment regimens including a combination of anti-CD38 mAbs with proteasome inhibitors (PIs) and/or immunomodulatory drugs (IMiDs) and dexamethasone. Relapsed and/or refractory MM (RRMM) patients may receive new immunotherapeutic approaches such as chimeric antigen receptor T-cell (CAR-T), bispecific T-cell engagers (3). Together, these therapeutic approaches have allowed an increase in the survival of MM patients.

Nevertheless, the genomic features of tumor cells and various interactions with the BM microenvironment make MM incurable, and relapse is a common issue for MM patients. MM is characterized by changes in BM microenvironment composition. BM microenvironment is composed of several cell types such as hematopoietic cells, mesenchymal stem cells, mesenchymal stromal cells (MSCs), osteoblast, osteoclast (OCs), endothelial cell, fibroblast, and immune cells (4). Among the immune cells, those most involved in the development of MM are various immunosuppressive cells, including myeloid-derived suppressor cells (MDSCs), T-cells, regulatory T-cells (T_regs_), regulatory B-cells (B_regs_), natural killer cells (NK) and tumor-associated macrophages (TAMs) (5).

In addition, the MM BM microenvironment represents an ideal niche because, through the release of growth factors and cytokines, it interacts with MM cells, promoting their proliferation and survival (6).

## 2 BM microenvironment composition in multiple myeloma and its involvement in immunotherapy resistance

**Figure 1:**
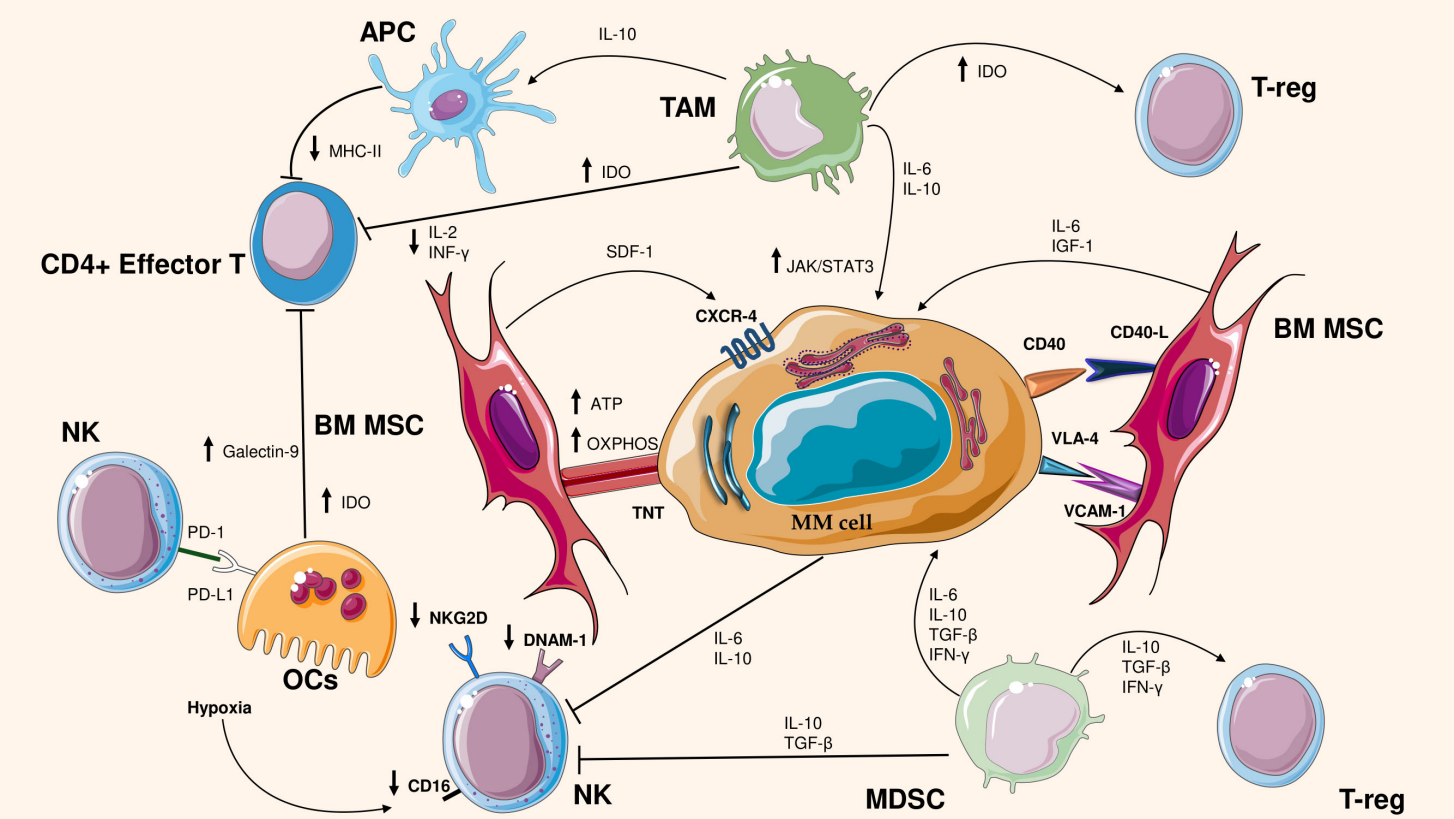
Cross talk between MM cells and bone microenvironment cells. The figure illustrates the interactions between cells in the bone marrow (BM) microenvironment and multiple myeloma (MM) cells, highlighting the mechanisms of immunosuppression and tumor support. Bone marrow stromal cells (BM MSCs) promote MM cell survival through mitochondrial transfer and pro-tumor signals (ATP, OXPHOS, IGF-1, IL-6). Myeloma cells interact with immunosuppressive cells such as tumor-associated macrophages (TAMs), regulatory T lymphocytes (T-reg) and myeloid suppressor cells (MDSCs), Natural Killer (NK), which secrete immunosuppressive cytokines (IL-10, TGF-β, IFN-γ). In addition, in MM occurs the reduced activity of immune effector cells, such as CD4+ T lymphocytes and NK cells, by decreasing the expression of key molecules such as MHC-II and NKG2D. Moreover, Osteoclasts (OCs) produce Galectin-9, IDO and affect the activity of NK through PD-L1/PD1 axis. These mechanisms contribute to immune evasion and disease progression.

The BM microenvironment is a dynamic and interactive ecosystem that plays a pivotal role in regulating the behavior of clonal PCs. In fact, BM microenvironment strongly influences the response of PCs to MM drug treatments. One of the main issues that influences survival or drug resistance is a tight crosstalk between PCs and the BM microenvironment. **Figure 1** summarizes the interactions between tumor microenvironment and MM cells. Below, we will discuss the microenvironment changes in MM that ensure tumor progression and an immunosuppressive environment.

### 2.1 Mesenchymal stromal cells

The BM microenvironment comprises MSCs and immune cells, which influence response. MSCs are cells exhibiting stemness activity and exhibit two basic properties: self-renewal and differentiation into various cell types. The first characteristic determines the ability to generate a daughter cell with the same stemness characteristics as the parent cell, the second feature allows MSCs to differentiate and generate adipose, cartilage, and bone tissue (7). Interaction between MSCs and PCs occurs through members of the integrin family, including syndecan-1 (CD138), CD44, vascular cell adhesion molecule 1 (VCAM1), lymphocyte function-associated antigen 1 (LFA-1), mucin 1 (MUC-1), intercellular adhesion molecule 1 (ICAM-1), very late antigen-4 (VLA-4) (ɑ4β1), and VLA-5 (8). VLA-4 is highly expressed on MM cells and is the only integrin able to mediate both PCs-extracellular matrix and PCs-BM MSCs interactions via separate binding sites (8).

PCs are also able to reach the BM microenvironment through the expression on their membrane of C-X-C chemokine receptor type 4 (CXCR-4). CXCR-4 creates an axis by binding with C-X-C motif chemokine ligand 12 (CXCL-12), which is a chemokine secreted by BM MSCs (9). Roccaro et al. demonstrated, by immunohistochemistry, that BM in which PCs are present show increased expression of CXCL-12 [also known as stromal cell-derived factor 1 (SDF-1)], compared to samples from healthy controls or patients with monoclonal gammopathy of undetermined significance (MGUS), which showed minimal and low expression of SDF-1 (9). They also showed that *in vivo* neutralization of SDF-1 results in a less receptive microenvironment for MM cells and reduces the homing and growth of MM cells (9). Furthermore, CXCL-12 upregulates VLA-4, which modifies the adhesion of PCs to MSCs and the secretion of cytokines by MSCs (4).

In a further study, performed in 2023, it was reported that the secretome of healthy MSCs was altered by priming MSCs, i.e. by culturing with MM cells, and that the overall secretome functionality changed from promoting MM cell quiescence to stimulating MM cell proliferation (10). They identified several dormancy-associated pathways that were suppressed by primed conditioned medium (CM), leading to the up-regulation of genes involved in the cell cycle, DNA damage repair, and proliferation. Among these pathways, they further explored the mTOR pathway. They proved that insulin-like growth factor type 1 (IGF1) induces MM cell growth, and that primed CM reduced the expression of RPTOR independent Companion Of mTOR Complex 2 (RICTOR), which is part of the mTOR2 pathway that contributes to shifting MM cells towards a proliferative state (10).

A new mechanism involved in drug resistance was discovered a few years ago, namely Mitochondrial Transfer. Mitochondrial transfer is based on communication between a donor and a receiving cell and can be regulated by different structures, such as extracellular vesicles, tunneling nanotubes (TNTs), and communicating junctions (11). TNTs are long-distance intercellular connections that allow the exchange between cells, of ions, and small molecules or the incorporation of mitochondrial genes or the mitochondria themselves into a recipient cell (11). Acquiring mitochondria via TNTs enhances the growth potential of tumor cells, provides survival benefits, and increases oxidative phosphorylation activity (OXPHOS) and the adenosine triphosphate (ATP) level of tumor cells. In addition to this, it improves their migratory properties and increases the possibility of developing resistance to chemotherapeutic treatment (12–14). Regarding this mechanism of drug resistance involving the acquisition of mitochondria, Matula et al. carried out a study in which primary MM cells and autologous BM-MSCs were used (15). The work aimed to achieve a more detailed comprehension of the mechanism by which MSCs protect MM from the cytotoxic action of chemotherapeutic drugs and therapeutic antibodies used in the treatment of MM. In fact, they treated the co-culture of BM-MSC and MM cells with several drugs, finding that BM-MSCs prevent MM from drug-induced cytotoxicity, since all drugs increased the uptake of BM-MSC-derived mitochondria by MM. Moreover, it was found that there was a correlation between the survival of MM, the drug concentration added, and the BM-MSC-derived mitochondrial incorporation of surviving MM cells. This suggests that the mitochondria derived from BM-MSCs worked as a survival signal for the MM cells and were more resistant to the cytotoxic effect of the drugs used (15).

### 2.2 Myeloid-derived suppressor cells

MDSCs are cells of neutrophil and monocyte lineages with potent immunosuppressive activity. In recent years, their role has emerged because several studies proved their involvement in immunosuppressing anti-tumor activity. The human MDSCs are less defined, lacking a Gr1 homologous. Commonly, MDSCs are defined as CD11b+ CD33+ HLA-DRlow/− cells and that do not express markers of mature myeloid or lymphoid cells (16). Among their different roles, these cells produce the enzyme arginase that depletes the environment of arginine, an essential amino acid for T-lymphocyte activity. In addition, they ensure the expansion of induced T_regs_ (17, 18). MDSCs can differentiate into TAMs and OCs. The presence of the latter underlies the characteristic bone disease observed in MM (19). MDSCs also have a remarkable ability to inhibit the activity of inducible nitric oxide synthase (iNOS), reactive oxygen species (ROS), and peroxynitrite (20). The consequence is to evade the immune system and to promote disease progression. Also, several soluble factors and cytokines contribute to the immunosuppressive activity of MDSCs in BM, such as interleukin-10 (IL-10), IL-6, transforming growth factor-β (TGF-β), CD40-CD40 ligand, and interferon-γ (IFN-γ). These cytokines contribute to the expansion of T_regs_ (18, 21).

### 2.3 Tumor-associated macrophages

An additional type of immune cell that is implicated in MM is TAM. Specifically, TAMs are an important population of macrophages that reside in large numbers at the tumor site and are strongly influenced by the tumor microenvironment (22). TAMs arise from circulating monocytes and are identifiable by the marker CD68. They are characterized by remarkable plasticity, in fact, after recruitment to the tumor site, they progressively acquire pro-tumor properties, making themselves similar to M2 macrophages (23). In MM they ensure proliferation and survival, angiogenesis, immunosuppression, and drug resistance (24). The tumor cell growth in MM, supported by TAMs, has been extensively studied in several articles. The factor behind this is the enhanced release of several cytokines, in particular IL-10 and IL-6, and the reduced secretion of IL-12 and tumor necrosis factor-α (TNF-α) (25). For example, *in vitro* data demonstrated that TAMs support MM cell survival through activation of the IL-6/JAK/STAT3 pathway. It has been shown by De Beule et al., that the co-culture of TAM with 5T33MM murine MM cells enabled the survival of myeloma cells, through the activation of the STAT3 pathway in 5T33MM cells (26). In addition, IL-10 ensures survival and proliferation of tumor cells in MM and the IL-10 production is regulated by IL-6 (27). IL-10 is also involved in angiogenesis. Indeed, IL-10 secreted by MM-associated TAMs in MM patients correlates positively with angiogenic cytokines such as vascular endothelial growth factor (VEGF) or angiopoietin-2 (Ang-2) (28). In addition to VEGF, macrophages can secrete other angiogenic factors such as C-C Motif Chemokine Ligand (CCL) and matrix metalloproteinase (MMP) (29).

In many cancer types, TAMs have been reported to influence the tumor microenvironment, leading to an immunosuppressive microenvironment and a reduced number of anti-tumor cells, such as CD8+ T-cells (30). Beider et al. demonstrated that MM-primed macrophages decreased T-cell proliferation and activation, through downregulation of IFN-γ secretion (31). In addition to this study, it was also demonstrated in single-cell RNA sequencing that mature CD14+ monocytes/macrophages change phenotypically, losing expression of major histocompatibility complex class II (MHC-II). This loss of expression results in immunosuppressive potential and suppressed T-cell activation (32). IL-10 also plays an important role in the expression of MHC-II. IL-10 has been proven to inhibit the MHC-II expression and the production of pro-inflammatory cytokines in antigen-presenting cells (APCs), which in turn limit the functions of effector T-cells (33). Another important factor, involved in immunosuppression, is Indoleamine 2,3-dioxygenase (IDO). IDO is an enzyme that degrades the essential amino acid tryptophan into kynurenine. IDO production is under activity of IL-32, a proinflammatory cytokine. In MM it has been shown that IL-32 is overexpressed in the BM and peripheral blood (PB) of MM patients. High expression of IL-32 stimulates IDO production in macrophages, this led to an inhibition of CD4+ T-cell growth, IL-2, IFN-γ, and TNF-α production. The result is a reduced immunogenic response. Additionally, IDO promotes T_regs_ differentiation (34).

### 2.4 Natural killer cells

NK cells have an impact on cancer due to their natural tumor suppressor potential. They are present in the BM, liver, spleen, lungs, uterus, thymus, and secondary lymphoid tissues (35). In MM patients, significant changes in NK subpopulation distribution and NK cell activity have been identified (36). NK cells have several inhibitory and activating receptors and their functionality depends on the balance between inhibitory and activating signals induced by interaction with their respective ligands (37). Among the activating receptors, one of the most important is NKG2D. Preclinical studies, in MM, have shown that some microvesicles induce the downregulation of NKG2D and transfer of NKG2DL to the surface of cells after internalization by NK cells. Thereafter, the NKG2D-NKG2DL axis mediates the NK cell fratricide (38). Moreover, Seymour et al. revealed a significant decrease in the NK cell activating receptor such as natural cytotoxicity triggering receptor 3 (NCR3), NKG2D, 2B4, and DNAX Accessory Molecule-1 (DNAM-1) and upregulation of the inhibitory receptor programmed death 1 (PD-1) in MM patients (39). In addition to this, in a preclinical study, hypoxia decreased NKG2D and CD16 expression in NK cells and impaired NK cell degranulation (40). Furthermore, Daly et al. demonstrated inhibition of cytotoxicity and cytokine production in NK cells *in vitro*. This happens because the sialic acid-like immunoglobulin (Siglec) (PSGL-1/CD43) of MM cells binds to the inhibitory Siglec-7 of NK cells (41).

NK cells also express chemotactic receptors, such as CXCR-1, CXCR-3, CXCR-4, CXCR-6, CX3CR-1, sphingosine 1-phosphate receptor 5 (S1P5), CCRL-2. Among these, CXCR-4 is worth mentioning. Downregulation of the CXCL-12 and its ligand CXCR-4 influences NK-cell trafficking in the BM and diminishes antitumor immune responses in MM patients, causing migration of NK cells outside of the BM (42). IL-6 and IL-10 levels, which are known to promote PCs proliferation, also promote the development of the NK-resistant tumor phenotype by inhibiting their activity (43).

### 2.5 Osteoclast

OCs are specialized multinucleated cells which are responsible for bone resorption, a key process in skeletal remodeling, repair and calcium homeostasis. OC differentiation originates from hematopoietic progenitors of the monocyte/macrophage lineage (44). This process is controlled by two cytokines: macrophage colony-stimulating factor (M-CSF) and nuclear factor kappa-Β activator receptor ligand (RANKL) (45, 46).

OCs play a fundamental role in the pathogenesis of bone disease, detectable in about 80% of patients with MM (47). In addition, OCs may regulate the immune system. In fact, bone resorption regulated by OCs is associated with immune activation of T-cells in autoimmune diseases. This is accomplished by crosstalk between OCs and T-cells (48).

A previous study suggests that OCs could serve as APCs (48). An et al. demonstrated the upregulation of immune-checkpoint molecules on OCs following the observation that OCs inhibit T-cell proliferation (49). They found high PD-L1 expression in OCs, higher in OCs than in PCs. The expression of PD-L1 could worsen immune inhibition by enhancing the binding of PD-1 on T-cells. Among the immunosuppressive molecules, they also evaluated the expression of CD200 and herpesvirus entry mediator (HVEM), both upregulated in OCs. CD200 is a membrane glycoprotein that mediates an immune regulatory signal through CD200R to suppress T and NK immune responses (49). They also showed IDO production during OCs formation and higher IDO production in OCs compared with PCs from the same patient samples. In addition, they found high secretion of galectin-9, a negative regulator of T helper 1 cell response, in the supernatant of OCs compared with monocytes (49). They also verified the high levels of galectin-9 in the BM of patients with MM compared with the serum of healthy donors. In this study, they explored the role of proliferation-inducing ligand A (APRIL), which is highly expressed in OCs. In addition, they performed transwell experiments to establish whether OCs modulate PD-L1 expression on MM cell lines via an APRIL-dependent manner. They discovered that PD-L1 expression increased in MM cell lines, through the MEK/ERK pathway, when they were cocultured with OCs (49).

Furthermore, Tai et al. confirmed that the OCs, which express APRIL and PD-L1, stimulate T_regs_ to suppress the proliferation of conventional T-cells. In fact, by combining blocking receptor/ligand axis mAbs, as anti-APRIL mAbs and -PD1/PD-L1-PD1/PD-L1 mAbs, this effect was overcome (48). Moreover, it has been shown that during osteoclastogenesis, CD38 expression is also induced (49). In this study, it was also shown that the use of isatuximab (Isa), the anti-CD38 mAb, significantly reduced the expression of CD38 on OCs, and suppressive function of T-cells by OC is attenuated (49). The reduction in CD38 is probably due to internalization of the target after mAb binding, a feature of Isa (50).

## 3 Immunotherapy resistance in multiple myeloma

As we have discussed above, immune dysfunction plays an important role in MM and drug resistance.

**Figure 2:**
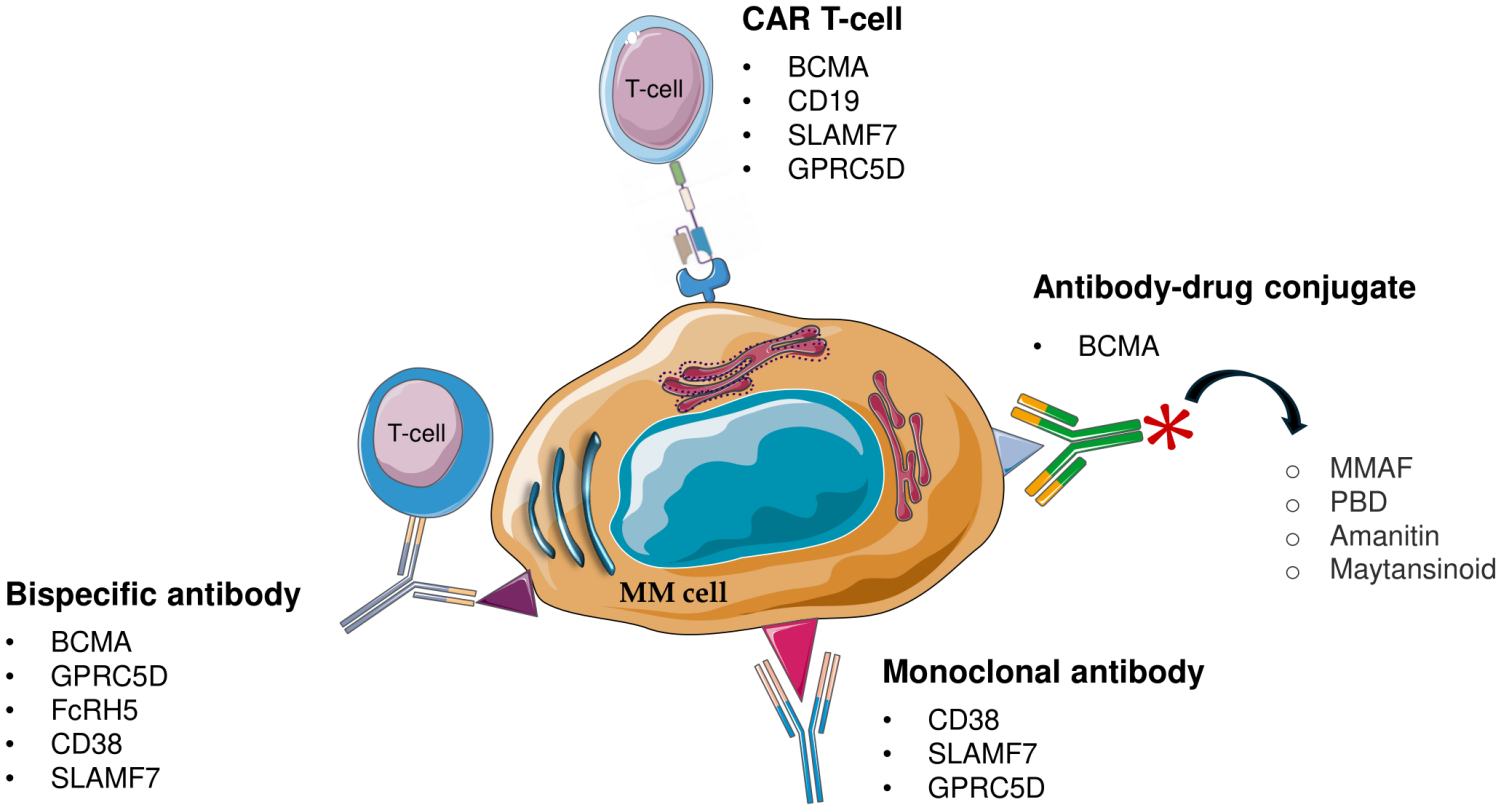
Schematic representation of immunotherapeutic approach for multiple myeloma (MM). MM, Multiple Myeloma; CAR T-cell, chimeric antigen receptor T-cell; BCMA, B-cell maturation antigen; GPRC5D, G protein-coupled receptor class C group 5 member D; FcRH5, Fc receptor-homolog 5; MMAF, monomethyl auristatin F; PBD, pyrrolobenzodiazepine.

Immunotherapy is an essential tool in the management of MM, giving hope to RRMM patients. There are nowadays several types of drugs that harness the immune system. Current immunotherapy is based on the use of mAbs, CAR-T immunotherapy, CAR-NK cells, antibody-drug conjugates, checkpoint-blocking antibodies, and bispecific antibodies (51) (**Figure 2**).

### 3.1 Monoclonal antibody resistance

mAbs include daratumumab [anti-CD38 (Dara)], elotuzumab [anti-signaling lymphocytic activation molecule F7 (SLAMF7) (Elo)], and Isa [anti-CD38 (Isa)]. The indirect mechanism of action of mAbs is similar and includes complement-dependent cytotoxicity (CDC), antibody-dependent cell-mediated cytotoxicity (ADCC) and antibody-dependent cell-mediated phagocytosis (ADCP).

CDC is activated by binding the mAbs to CD38 expressed on the cell surface and subsequent recruitment of C1q protein to the Fc domain of the mAbs. This event triggers the complement cascade, resulting in the formation of the membrane attack complex (MAC), which is responsible for pore formation on the plasma membrane and direct lysis of tumor cells (52).

ADCC is induced by NK cells, which, when activated, release perforins and granzymes, resulting in target cell death. Lysis of MM cells treated with anti-CD38 mAbs is generally dose-dependent (52).

ADCP is mediated by the interaction between the Fc domain of the mAbs and Fc gamma receptors (FcγR) on monocytes and macrophages. This binding promotes the phagocytosis of opsonized tumor cells (52).

Special mention should be made of Dara and Isa, both of which target CD38 but binding different epitopes that could result in slight mechanisms. Dara is a human immunoglobulin G1-kappa (IgG1k) mAb directed against the cell surface glycoprotein CD38, instead Isa (formerly SAR650984) is a chimeric humanized, IgG1-derived mAb. These mAbs bind two different epitopes on CD38, in fact, the binding of Dara induces a structural breakthrough in the C-terminal region of CD38, which is not noted in the complex with Isa (53).

Dara exerts its function mainly through CDC (54) but Isa uniquely induces direct cell death without cross-linking agents (55). In contrast to Dara, the antitumor activity of Isa relies more heavily on ADCC than CDC (56). Given that CD38 has multiple functions as a receptor and enzyme, in several studies it was analyzed how mAbs impact the enzymatic activity. Indeed, Isa inhibits both CD38 hydrolase and cyclase activity, while Dara only partially inhibits cyclase activity and enhances hydrolase activity (52). In MM, a particular category of patients is those with more copies of chromosome 1q21. This group of patients has a reduced response to treatment with Dara compared to treatment with Isa (57, 58). There is still no strong scientific evidence for this issue, but what is hypothesized is increased expression of CD55, which is a gene located on chromosome 1, and which is over-expressed during disease progression in patients treated with Dara (56, 59). CD55 has been shown to prevent CDC (60), which is the main mechanism of action of Dara (52), unlike Isa. In addition, CD46, located on chromosome 1, is also a complement regulator (52). Another study that shed more light on this issue, conducted by Ogiya et al., showed that BM MSCs produce IL-6, which binds to its receptor IL-6R on myeloma cells and this causes CD38 downregulation via the JAK-STAT3 pathway (61). The interesting point is that the IL-6R gene is on chromosome 1q21.

Furthermore, since CD38 also shows enzymatic activity involved in adenosine production, CD38 mAbs may also inhibit adenosine production and the function of adhesion molecules. CD38 mAbs can also induce immunomodulatory cells to suppress the inhibitory effect of MM cells on effector T-cells, thus activating T cells to kill tumour cells (62, 63). Elo affects MM mainly by direct activation of NK cells and mediating ADCC through the CD16 pathway (64).

MM cells can evade mAbs-based immunotherapy through resistance mechanisms. Dara targets MM cells by binding CD38, but low CD38 expression is linked to resistance (59). Nijhof et al. observed that non-responders exhibit low baseline CD38 levels and that CD38 expression decreases further during treatment, affecting both non-responders and partial responders (59). There are different assumptions about the reduction of CD38. One of these concerns is Dara’s function to deplete MM cells with high CD38 expression. After depletion, clones with low CD38 expression expand, making patients unresponsive to Dara treatment (65). The CD38 depletion induced by Dara involves not only MM cells but also CD38+ expressing immune cells, including NK, B, and T cells. It was noted that Dara treatment induces depletion of PB and BM NK cells by fratricidal ADCC against CD38+ NK cells, while CD38- NK survive (66). In two trials, GEN501 and SIRIUS, patients’ NK cells were analyzed and levels of these cells decreased immediately after the first infusion of the drug (59). This effect can strongly influence NK-mediated ADCC, reducing the efficacy of Dara and increasing the risk of relapse (67).

In addition, the BM microenvironment protects the MM cell from ADCC mediated by Dara. Concerning this, De Haart et al. (68) demonstrated the overexpression of the anti-apoptotic protein Survivin in MM cells upon interaction with BM. Subsequently, they tested the sensitivity of MM cells to Dara-dependent ADCC in the absence/presence of BM MSC and in the absence/presence of the YM155 molecule that efficiently suppresses survivin expression in tumor cells. Co-culture treatment of MM/BM MSC YM155 increased Dara-mediated ADCC by overcoming the BM microenvironment’s protective role against Dara treatment (68).

Besides ADCC, myeloma cells can also evade ADCP through the upregulation of CD47. The upregulation of CD47 was demonstrated by Sun et al. (69) who found that CD47 gene expression is directly correlated to the stage of the disease. Notably, PCs from MM patients overexpress CD47 compared to those from MGUS, which have a higher expression than healthy subjects (69). CD47 binds the signal regulatory protein alpha (SIRPα) on TAMs. The CD47/SIRPα complex acts as a ‘don’t eat me’ signal resulting in a blockade of TAM activity (70).

The phenomenon of mitochondrial trafficking promoting bioenergetic plasticity in MM was also investigated regarding the CD38 molecule (71). In this study published in 2019, it was shown that CD38 is required for the formation of TNTs that facilitate protumor mitochondrial transfer in MM. They also observed increased levels of apoptosis in MM cells when the number of mitochondria transferred was reduced. shRNA-mediated knockdown of CD38 inhibited mitochondrial transfer and TNT formation *in vitro*, blocked mitochondrial transfer and improved animal survival *in vivo* (71) (**Table 1**).

**Table 1:** Mechanisms of resistance to immunotherapies in multiple myeloma.

Immunotherapy type	Mechanism of action	Mechanisms of resistance
mAbs: • Dara and Isa anti CD38 • Elo anti SLAMF7 *via* CD16	- ADCC (Antibody-Mediated Cellular Cytotoxicity) - CDC (Complement-Dependent Cytotoxicity) - ADCP (Antibody-Mediated Cellular Phagocytosis)	-Downregulation of target antigen (CD38 for Dara and Isa, SLAMF7 for Elo) - Upregulation of CD47 which blocks phagocytosis - Depletion of immune effector cells (NK, macrophages) - Interference of the bone marrow (BM) microenvironment (TNT formation)
Antibody-Drug Conjugates (MMAF, PBD, amanitine)	- Downregulation of target antigen (BCMA loss) - Increased expression of efflux pumps (ABC transporters) that remove the drug from the cell - Resistance to cytotoxic effects of payload	- Reduced drug efficacy - Primary or acquired resistance - Survival of MM cells despite treatment
CAR-T cells (BCMA, GPRC5D, SLAMF7)	- Antigen recognition and T-cell activation to kill multiple myeloma (MM) cells	- Antigen escape: downregulation/mutation of BCMA/GPRC5D - T-cell exhaustion (loss of function over time) - Immunosuppression in the BM (TGF-β, IL-10, MDSCs) - Expansion of immunosuppressive cells (T_regs_, TAMs)
Bispecific Antibodies (BCMAxCD3, GPRC5DxCD3, FcRH5xCD3)	- Simultaneous recognition of T-cell and MM cell for lysis of tumoral cell	- T-cell depletion during treatment - T-cell depletion (increase in PD-1, TIGIT, TIM-3) - Immunosuppression in the BM (TGF-β, IL-6, IL-10) - Loss of target antigen expression (BCMA loss)

In this class of drugs, the ones to be included are also the antibody-drug conjugates (ADCs), used in the treatment of patients with RRMM who have received at least 4 lines of therapy. ADCs allow the delivery of potent anti-cancer drugs directly to MM cells, helping the immune system to target tumor cells. Circulating ADCs bind to target antigens on myeloma cells through their mAb, leading to ADC internalization (72). Once inside the cell, ADCs are degraded in lysosomes, releasing the conjugated cytotoxic payload. The released toxic payload induces DNA damage in the nucleus and/or disrupts microtubule polymerization and function in the cytoplasm, ultimately triggering apoptosis (72). ADC covalently bind a cytotoxic drug that can be monomethyl auristatin F (MMAF) through a non-cleavable maleimidocaproyl (mc) linker (73). MMAF inhibits tubulin polymerisation and induces G2-M growth arrest, thus causing caspase 3/7-dependent apoptosis (73).

In addition to MMAF, ADCs have been engineered to bind other drugs such as pyrrolobenzodiazepine (PBD) or amanitine (74), which prevents the transcription process by inhibiting RNA polymerase II (74). A third ADC is the non-cleavable maytansinoid (74). Also, concerning ADCs, resistance mechanisms may arise due to low expression of the antigen to which they bind. This is the case with B-cell maturation antigen (BCMA), because as seen above, MM patients may present downregulation, loss or mutations of BCMA (75).

Another resistance mechanism that could affect the action of ADCs is the type of drug that is used. This is because MM cells may have ATP binding cassette (ABC) transporters on their surface (74). These transporters could recognize drugs as their substrates and extrude them outside the cell and thus block their cytotoxic action. Therefore, a good strategy would be to conjugate antibodies with drugs that are not substrates of these transporters and overcome drug resistance (74).

### 3.2 CAR-T therapy resistance

A promising MM treatment is CAR-T-based therapy. CARs are fusion proteins engineered to target specific antigens which are expressed on the surface of cells. This therapy allows the reprogramming of T-cells to target myeloma cells. CARs are composed of an antigen recognition domain and a T-cell activation domain, usually CD3ζ. These two parts are linked via an extracellular spacer region and an element that crosses the transmembrane (76). To develop second-generation CARs, a costimulatory domain was introduced, such as CD28, 4-1BB, OX40, or ICOS. This domain is in close contact with the intracellular domain, resulting in greater anti-tumor activity of modified T-cells and greater efficacy than first-generation CARs lacking this domain (77). Most CAR-T cells currently evaluated in MM target several antigens, such as BCMA, CD19, SLAMF7, CD38, and G protein-coupled receptor class C group 5 member D (GPRC5D) (78).

Concerning BCMA CAR-T, there are two types of drugs that target BCMA. The first is Idecabtagene vicleucel (ide-cel), approved in 2021, whereas the second is Ciltacabtagene autoleucel (cilta-cel), approved in 2022 (79). Although they are two CAR-T anti-BCMA, they have different mechanisms. Indeed, ide-cel contains a single mouse-derived binding domain to target only one epitope of the BCMA antigen, whereas cilta-cel expresses two camelid heavy chains (VH) of mAbs to bind with two separate epitopes of BCMA antigen. This actually renders cilta-cel a unique CAR-T cell agent that provides higher avidity of binding to target cells, higher activity, and lower immunogenicity than ide-cel (79).

The efficacy of cilta-cel was investigated in the CARTITUDE-1 clinical trial, which found that one-third of patients remain in remission for ≥5 years after a single infusion of cilta-cel without maintenance therapy. This highlights an excellent outcome given the historically poor prognosis for RRMM patients with an OS of around 1 year. In addition, progression-free patients had a fitter immune T-cell phenotype and a higher E:T ratio at peak expansion (80).

The efficacy of ide-cel, instead, was evaluated in KarMMa clinical trial, underlying significantly longer progression-free survival than was seen with standard regimens, and responses were deeper (81).

An important aspect in the context of CAR-T use, is the assessment of tumor burden. Indeed, in CARTITUDE-1 lower tumor burden at baseline was associated with progression-free status at ≥5 years (80). In addition, it has also been shown that patients without extramedullary disease respond better to cilta-cel therapy than patients with extramedullary disease (82). Even in the KarMMa trial there was a trend toward a moderately lower complete response rate in patients with a high disease burden (≥50% MM cells located in the BM) compared with patients with a relatively low tumor burden (83).

Although CAR-T therapy has shown encouraging evidence in terms of efficacy, the resistance challenge is still a considerable problem that needs to be overcome. The BM microenvironment can create a suppressive effect on CAR-T through the secretion of immunosuppressive cytokines, such as TGF-β and IL-10, as well as the recruitment of T_regs_ and MDSCs.

Leblay et al. performed a single-cell analysis on the immunophenotypic and transcriptomic characterization of BM T-cells from sensitive and resistant MM patients treated with BCMA CAR-T cell therapy. They found, through cellular indexing of transcriptomes and epitopes by sequencing (CITE-seq), an enrichment of CD4+ T-cells with a higher CD4/CD8 ratio in responding patients. Phenotypic (CD45RA, CD45RO, CD95, CCR7, CD62L, CD28, CD27) and transcriptional (TCF7, LEF1, GATA3, EOMES, TBX21, PRDM1) signatures also identified a higher proportion of memory-like T-cells (Tscm, Tcm) in responding patients. In contrast, T-cells of resistant patients were enriched in terminally exhausted (Tex) and senescent cells with loss of CD28, elevated levels of GMZH and GMZB, CD57+, CD69+, and CD160+, as well as upregulation of TBX21. The expression of T-cell checkpoint inhibitors, such as LAG3, TIGIT, and PD1, was elevated in these Tex cells and some T effector memory (Tem) (84).

In 2021, Holthof et al. tested a panel of 10 BCMA-, CD38-, and CD138-specific CAR-T cells with different affinities against a UM9 MM cell line and patient-derived MM cells in the presence versus absence of BM MSCs (85). They observed a comparable association between the level of the lytic capacity of CAR-T cells in the absence of BM MSCs and the inhibitory effect of BM MSCs in this *ex vivo* context (85). Furthermore, they demonstrated through *in vivo* experiments that BM MSCs-mediated resistance against CAR-T cells was effectively modulated by FL118, an inhibitor of the anti-apoptotic proteins Survivin, Mcl-1, and XIAP (85).

In 2023, Li et al. performed an in-depth analysis of the mechanisms of BCMA CAR-T treatment resistance by single-cell RNA sequencing of PCs and BM immune cells (86). Even if the patient numbers were low, they reported that the percentage of depleted CD8+ effector T-cells increased in relapsed patients after BCMA CAR-T treatment, compared to the percentage at baseline. IFN-responsive CD8+ effector T-cells also increased significantly in relapsed patients after treatment with BCMA CAR-T cells, who also had exhausted phenotypes (86). They also showed an increase in the proportion of monocytes/macrophages at the time of relapse after BCMA CAR-T cell therapy. Monocytes/macrophages showed tumor-promoting phenotypes and induced T-cell depletion in RRMM patients at the time of progression (86). In addition, they performed cell-cell communication analysis, which showed that monocytes/macrophages are key players in relapse after BCMA CAR-T cell therapy. Indeed, the monocyte/macrophage signaling pathways identified include APRIL, MIF, RESISTIN, BAFF, ITGB2, CLEC, and CD99. Monocyte/macrophage entry signaling pathways at progression include MIF, CD99, ITGB2, CCL, CSF, IL-4 and IL-2 (86). They analyzed the heterogeneity of NK cells and DC cells in MM patients at baseline and progression. Their investigation showed that the proportions of TIGIT+ and/or CD69+ NK cells were significantly higher in patients who relapsed after BCMA CAR-T cell therapy (86). TIGIT is a checkpoint receptor that is considered to be involved in mediating NK-cell depletion in tumors (87). Concerning DC cells, they observed an increase in the percentages of the ISG15+ DC subpopulation at progression (86). Several studies have reported that ISG15 induces the expression of E-cadherin in DCs *in vitro*, an adhesion molecule whose expression can prevent DC mobility and serve as an escape mechanism for several tumors (88).

Sakemura et al. examined the impact of cancer-associated fibroblasts (CAFs) on the efficacy of CAR-T cells (89). They showed that CAFs, isolated from the BM of patients, promote MM growth and inhibit BCMA CAR-T cells. Furthermore, CAFs suppress CAR-T cells through both contact-dependent and cytokine-mediated effects. Indeed, when BCMA CAR-T cells were stimulated and co-cultured with BM CAFs, the surface expression of inhibitory receptors such as PD-1 was significantly increased on CAR-T cells, while BM CAFs simultaneously overexpressed inhibitory ligands such as PD-L1 (89).

In addition, a study in 2024 was conducted to evaluate the ability of TAMs to inhibit the BCMA CAR-T-mediated MM cell killing *in vitro*. Single cell analysis in both human and murine identified C1qb ligand in macrophages with C1qbp in the tumor among the major interactions. BM-derived macrophages, after stimulation/polarization with MM lines, strongly inhibited the *in vitro* cytotoxic activity of anti-BCMA CAR-T cells. In addition, C1q+ macrophages showed upregulation of markers such as transmembrane immune signaling adaptor TYROBP (TYROBP) and Fc epsilon receptor Ig (FCER1G), which are associated with polarization and infiltration of macrophages (90).

As mentioned above, in addition to supporting the growth of MM cells, TGF-β can contribute to immunosuppressive conditions in the BM microenvironment, allowing MM cells to escape the immune response. In an intriguing study from 2022, Alabanza et al. (91) designed a novel BCMA CAR that co-expresses the dominant negative form of the TGF-β type 2 receptor, B2ARM, in order to confer resistance to CAR-T cells from the suppressive effects of TGF-β, which is widely stored in the BM microenvironment. B2ARM CAR-T cells had robust proliferation and cytotoxicity even after prolonged treatment with exogenous TGF-β, which has suppressive activity (91). To evaluate the efficacy of B2ARM CAR-T cells *in vivo*, they used intradermal xenograft models of tumor cells in NSG mice. Armored B2ARM CAR-T cells successfully eradicated tumors. In addition, B2ARM CAR T-cells demonstrated enhanced cytokine and granzyme B production and mediated increased target cell killing. The design of armored B2ARM CAR-T cells may contribute to overcoming the limitations of current BCMA CAR-T cell therapies and dominate the tumor-suppressive MM microenvironment (91) (**Table 1**).

### 3.3 Bispecific T-cell engagers resistance

To date, therapy based on Bispecific antibodies (BsAbs), also known as Bs T-cell engagers (TCE), is also being developed, which is showing promising results in RRMM patients. These antibodies are able to simultaneously target two antigens, generally the CD3 molecule of T-cells and the antigen of the tumor cell (92). The BsAbs, currently approved and under investigation for MM are directed against BCMA (teclistamab and elranatamab), GPRC5D (talquetamab), the homolog of the Fc 5 receptor (FcRH5) and CD38 on PCs. In addition to these, other BsAbs directed against SLAMF7 and CD138 were also engineered.

MM, however, is highly aggressive and despite favorable effects, almost one-third of patients do not respond to BsAbs therapy (primary resistance). In addition, most responding patients treated with BsAbs will eventually develop disease progression (acquired resistance) (93).

This resistance may be caused by intrinsic factors such as loss of the BCMA antigen due to homozygous deletion of the Tumour Necrosis Factor Receptor Superfamily Member 17 (TNFRSF17) gene encoding the BCMA protein (94), or biallelic inactivation of GPRC5D due to a homozygous deletion or a monoallelic deletion with mutation (1 frameshift indel, 1 missense and 2 nonsense mutations) (95).

In addition to intrinsic factors, extrinsic factors, linked to the microenvironment, also play a crucial role. The response of tumor cells to BsAbs treatment is influenced by a variety of factors outside the tumor, such as the pre-existing T-cell profile, its evolution over time, and the immunosuppressive environment induced by the MM cells and previous treatments. Verkleij et al., in a preclinical study, observed that the capacity of talquetamab to kill MM cells is reduced when there is a high proportion of certain T-cell populations, including those expressing the depletion marker PD-1, activated T-cells expressing HLA-DR and T_regs_ (96). In the Vk*MYC mouse model of transplantable MM, treatment with Bs TCE anti-BCMAxCD3 led to an upregulation of PD-1 in T-cells, which showed reduced functionality over time, causing relapses (97). Friedrich et al. showed that the accumulation of exhausted CD8+ clones is a predictor of treatment failure with anti-BCMAxCD3 TCE in MM patients (98). Similarly, several basic immune factors have been identified that suggest a probable negative response to TCE, such as a significant increase in T-cells expressing exhaustion markers (PD-1, TIGIT, and TIM-3) during BsAbs treatment, accompanied by a reduced proliferative potential, diminished cytokine secretion, and impaired antitumor activity (99). They also observed poor activity of BsAbs in samples with high T_regs_ numbers and a low T-cell/MM cell ratio (99). These results emphasize the role of the T-cell repertoire in determining the response to Bs TCE therapy. Other factors contribute to the immunosuppressive environment in MM, promoting resistance to TCE. These include the interaction between myeloma cells and BM MSCs, as well as the presence of inhibitory cytokines such as TGF-β, IL-6, and IL-10, and myeloid cells (100, 101). The interaction between MM cells and BM MSCs has been shown to increase the resistance of tumor cells to T-cell-mediated cytotoxicity (102). In *in vitro* experiments, the addition of BM MSCs reduced the efficacy of talquetamab in killing MM cells, an effect mediated by direct cell-to-cell interaction, but not by the soluble factors secreted by BM MSCs, suggesting the activation of intrinsic resistance mechanisms in tumor cells (96). Furthermore, immunosuppressive myeloid cells, such as MDSCs and plasmacytoid DC, have been shown to contribute to an environment that promotes myeloma progression (103–106) (**Table 1**).

## 4 Therapeutic approaches to overcome immunotherapy resistance

Overcoming resistance to immunotherapy is one of the most engaging challenges in the context of MM. For instance, one possible strategy to overcome resistance to CD38 mAbs could be to introduce the use of CD47 mAbs, thus blocking the CD47/SIRPα axis and allowing TAMs to perform their function (107). Several studies have shown promising preclinical results for anti-CD47 therapies in the treatment of hematological malignancies (107). Indeed, Storti et al. reported that treatment with Dara increases MM cell death, especially in the presence of a CD14+/CD16+ monocyte subset, and that the combination of Dara with anti-CD47 increases the killing of MM cells resistant to Dara alone (108). Since all-trans retinoic acid (ATRA) has been shown to reduce the expression of CD55 and CD59, potentiating the effect of Dara *in vitro* and in a mouse model, it is proposed as a strategy to enhance the effect of CD38 mAbs (109). Another one of the strategies already used is combining the use of CD38 mAbs with IMiDs, since IMiDs are able to induce NK cell activation and CD38 upregulation on MM cells, leading to a synergistic enhancement of the cytotoxic effects of CD38 mAbs (52). IMiDs have also demonstrated improvements in ADCC mediated by Dara in lenalidomide‐refractory MM cells, while pomalidomide enhances ADCC induced by Isa *in vitro* and *in vivo* (52).

A potential strategy could be targeting CD39 and CD73 in combination to reduce ADO production, which is involved in immunosuppression (110).

Interestingly, Chemlal et al. demonstrated a significant negative correlation between CD38 and Enhancer of Zeste Homolog 2 (EZH2) expression; indeed, the inhibition of EZH2 upregulates CD38 on surface and increases ADCC both in HMCLs and primary MM cells (111). In the context of EZH2, Liu et al. recently investigated the role of *KDM6A* (112). *KDM6A* is a histone demethylase that removes H3K27 trimethylation (H3K27me3), catalyzed by the EZH2-containing polycomb repressive complex 2 (PRC2). The loss or inactivation of *KDM6A* increased the level of H3K27me3, resulting in the downregulation of both CD38 and CD48 expression, which led to reduced ADCC. EZH2 inhibitors can therefore increase CD38 and CD48 expression and enhance Dara-mediated ADCC (112). In fact, CD48 is a ligand expressed on MM cells that binds with its receptor 2B4 on NK cells for their activation (112).

Concerning BCMA, it is cleaved from the MM cell surface by γ-secretases, resulting in diminished cellular expression and increased levels of soluble serum BCMA (sBCMA), a known adverse prognostic feature (113). Elevated levels of circulating sBCMA compromise the anti-BCMA antibody binding to MM cells *in vitro* (114). γ-secretase inhibition has been shown to increase BCMA expression in MM cells and reduce sBCMA *in vitro.* Recent studies have shown that the ubiquitin proteasome system degrades BCMA and that treatment with a proteasome inhibitor increases BCMA surface expression and improves BCMA CAR-T efficacy, justifying the combination of PI and BCMA-CAR-T in future studies (115).

One more strategy that could be used, and which has already been implemented in several clinical trials, is to target dual antigen. An example would be the dual target BCMA/CD19, even though CD19 is expressed by a very small part of the MM patient population (115). APRIL recognizes both BMCA and TACI, another MM epitope. AUTO2 is a CAR-T cell construct that incorporates a truncated form of APRIL as an antigen-binding domain, which allows dual targeting of BCMA and TACI (115). Additional bispecific CAR-Ts are in preclinical development, including a BCMA/CD24 CAR-T and a CAR-T directed against BCMA and MICA (human MHC class 1 related chain A gene), which is upregulated by MM cells as an immune evasion tool (115).

In addition, in a study performed in patients treated with cilta-cel, increased IL-15 production was found in the group with longer progression free survival (116). A BCMA CAR-T, designed to release soluble IL-15, demonstrated improved MM cell killing *in vitro* (115).

Sakemura et al. also developed CAR-Ts directed against both MM cells (BCMA) and CAFs (SLAMF7), proving enhanced functionality of CAR-Ts compared with BCMA CAR-Ts alone (89).

Another approach could be the use of IMiDs that stimulate upregulation of nuclear factor kappa-light-chain-enhancer of activated B cells (NFKB) expression (117) and increase CD8-positive T-cells and memory T-cells subsets (118).

The use BCMA-bispecific antibody in combination with a cereblone E3 ligase modulator (CelMod), mezigdomide, improved T-cell activation and cell killing in a preclinical model (115). In addition, IMiDs have been shown to boost BCMA CAR-T function *in vitro* (115).

Considering such features as extramedullary disease or tumor burden is an important aspect in the choice of therapy. Reproducing these issues *in vitro* is infeasible, so predicting a response to therapy is almost impossible, but a deeper investigation of tumor burdens and extramedullary disease role is essential in order to design more effective therapeutic strategies.

Regarding therapy based on CAR-Ts and BsAbs, treatment sequencing could be evaluated, as it seems that CAR-Ts should preferably be administered before BsAbs in the treatment regimen of eligible patients. In the MagnestisMM trials, patients treated with elranatamab, and previously exposed to BCMA CAR-T, had an overall response rate of 52.8%, compared with 61% in CAR-T naive patients (119, 120).

## 5 Conclusions

In the last few years, significant progress has been made in the development of immunotherapy to treat MM patients, particularly those with RRMM. Despite this, drug resistance remains a major challenge.

The short duration of remission and high relapse rate is an issue to be considered because it limits the long-term survival of MM patients. Mechanisms of resistance also affect immunotherapies. mAbs need to overcome antigen downregulation, CAR-T cells struggle with antigen escape, bispecific antibodies struggle with an immunosuppressive environment of the BM, and antibody-drug conjugates are limited by antigen loss and efflux mechanisms, even if for this last class of drugs there are still few studies investigating microenvironment resistance.

Future strategies should focus on overcoming these barriers through combinatorial approaches that target both MM cells and the BM microenvironment. Enhancing CAR-T cell persistence, the development of “armored” CAR-T cells capable of resisting immunosuppressive cytokines (e.g., TGF-β) or secreting immune-stimulatory factors (e.g., IL-15) represents a promising strategy to prolong persistence. Blocking immune checkpoints and counteracting stromal interactions may improve treatment efficacy. A deeper understanding of MM resistance mechanisms will be key to developing more durable therapeutic strategies.

Prospective studies are needed to overcome antigen escape. For example, since many RRMM patients lose BCMA expression following therapy targeting this antigen, it would be interesting to research other antigens that can be targeted, such as CD56, CD229, CCR10, CD44v6, GPRC5D, FcRH5, mucin 1 (MUC1), SLAMF7, TACI.

Another strategy could involve targeting metabolic crosstalk and organelle transfer between MM cells and BM components. Inhibition of mitochondrial trafficking, for example, can impair survival of MM cells and overcome drug resistance.

Although immunotherapies represent an effective and option for the treatment of MM patients, many open questions remain. In particular, it is critical to investigate strategies to optimize this therapy and improve its long-term outcomes.

## References

[1] Kawano Y Roccaro AM Ghobrial IM Azzi J. Multiple myeloma and the immune microenvironment. Curr Cancer Drug Targets. (2017) 17:806–18. doi: 10.2174/1568009617666170214102301, PMID: 28201978

[2] Shimizu K Iyoda T Yamasaki S Kadowaki N Tojo A Fujii AS. NK and NKT cell-mediated immune surveillance against hematological Malignancies. Cancers. (2020) 12(4):817. doi: 10.3390/cancers12040817, PMID: 32231116 PMC7226455

[3] Kumar SK Callander NS Adekola K Anderson LD Jr. Baljevic M Baz R. Multiple myeloma, version 2.2024, NCCN clinical practice guidelines in oncology. J Natl Compr Canc Netw. (2023) 21:1281–301. doi: 10.6004/jnccn.2023.0061, PMID: 38081133

[4] Iannozzi NT Marchica V Toscani D Burroughs Garcia J Giuliani N Storti P. Molecular features of the mesenchymal and osteoblastic cells in multiple myeloma. Int J Mol Sci. (2022) 23(24):15448. doi: 10.3390/ijms232415448, PMID: 36555090 PMC9779562

[5] Holthof LC Mutis T. Challenges for immunotherapy in multiple myeloma: bone marrow microenvironment-mediated immune suppression and immune resistance. Cancers. (2020) 12(4):988. doi: 10.3390/cancers12040988, PMID: 32316450 PMC7226482

[6] Garcia-Ortiz A Rodriguez-Garcia Y Encinas J Maroto-Martin E Castellano E Teixido J. The role of tumor microenvironment in multiple myeloma development and progression. Cancers. (2021) 13(2):217. doi: 10.3390/cancers13020217, PMID: 33435306 PMC7827690

[7] Ridge SM Sullivan FJ Glynn SA. Mesenchymal stem cells: key players in cancer progression. Mol Cancer. (2017) 16:31. doi: 10.1186/s12943-017-0597-8, PMID: 28148268 PMC5286812

[8] Di Marzo L Desantis V Solimando AG Ruggieri S Annese T Nico B. Microenvironment drug resistance in multiple myeloma: emerging new players. Oncotarget. (2016) 7:60698–711. doi: 10.18632/oncotarget.10849, PMID: 27474171 PMC5312413

[9] Roccaro AM Sacco A Purschke WG Moschetta M Buchner K Maasch C. SDF-1 inhibition targets the bone marrow niche for cancer therapy. Cell Rep. (2014) 9:118–28. doi: 10.1016/j.celrep.2014.08.042, PMID: 25263552 PMC4194173

[10] Mehdi SJ Ghatak K Ling W Johnson SK Epstein J Nookaew I. Growth and dormancy control of myeloma cells by mesenchymal stem cells. Leuk Res. (2023) 133:107355. doi: 10.1016/j.leukres.2023.107355, PMID: 37499483

[11] Torralba D Baixauli F Sanchez-Madrid F. Mitochondria know no boundaries: mechanisms and functions of intercellular mitochondrial transfer. Front Cell Dev Biol. (2016) 4:107. doi: 10.3389/fcell.2016.00107, PMID: 27734015 PMC5039171

[12] Caicedo A Fritz V Brondello JM Ayala M Dennemont I Abdellaoui N. MitoCeption as a new tool to assess the effects of mesenchymal stem/stromal cell mitochondria on cancer cell metabolism and function. Sci Rep. (2015) 5:9073. doi: 10.1038/srep09073, PMID: 25766410 PMC4358056

[13] Farge T Saland E de Toni F Aroua N Hosseini M Perry R. Chemotherapy-resistant human acute myeloid leukemia cells are not enriched for leukemic stem cells but require oxidative metabolism. Cancer Discov. (2017) 7:716–35. doi: 10.1158/2159-8290.CD-16-0441, PMID: 28416471 PMC5501738

[14] Henkenius K Greene BH Barckhausen C Hartmann R Marken M Kaiser T. Maintenance of cellular respiration indicates drug resistance in acute myeloid leukemia. Leuk Res. (2017) 62:56–63. doi: 10.1016/j.leukres.2017.09.021, PMID: 28985623

[15] Matula Z Mikala G Lukacsi S Matko J Kovacs T Monostori E. Stromal cells serve drug resistance for multiple myeloma via mitochondrial transfer: A study on primary myeloma and stromal cells. Cancers. (2021) 13(14):3461. doi: 10.3390/cancers13143461, PMID: 34298674 PMC8307863

[16] Gabrilovich DI Ostrand-Rosenberg S Bronte V. Coordinated regulation of myeloid cells by tumours. Nat Rev Immunol. (2012) 12:253–68. doi: 10.1038/nri3175, PMID: 22437938 PMC3587148

[17] Giallongo C Tibullo D Parrinello NL La Cava P Di Rosa M Bramanti V. Granulocyte-like myeloid derived suppressor cells (G-MDSC) are increased in multiple myeloma and are driven by dysfunctional mesenchymal stem cells (MSC). Oncotarget. (2016) 7:85764–75. doi: 10.18632/oncotarget.7969, PMID: 26967390 PMC5349872

[18] Malek E de Lima M Letterio JJ Kim BG Finke JH Driscoll JJ. Myeloid-derived suppressor cells: The green light for myeloma immune escape. Blood Rev. (2016) 30:341–8. doi: 10.1016/j.blre.2016.04.002, PMID: 27132116 PMC6411302

[19] Bolzoni M Toscani D Costa F Vicario E Aversa F Giuliani N. The link between bone microenvironment and immune cells in multiple myeloma: Emerging role of CD38. Immunol Lett. (2019) 205:65–70. doi: 10.1016/j.imlet.2018.04.007, PMID: 29702149

[20] Gabrilovich DI Nagaraj S. Myeloid-derived suppressor cells as regulators of the immune system. Nat Rev Immunol. (2009) 9:162–74. doi: 10.1038/nri2506, PMID: 19197294 PMC2828349

[21] Kuwahara-Ota S Shimura Y Steinebach C Isa R Yamaguchi J Nishiyama D. Lenalidomide and pomalidomide potently interfere with induction of myeloid-derived suppressor cells in multiple myeloma. Br J Haematol. (2020) 191:784–95. doi: 10.1111/bjh.16881, PMID: 32558939

[22] Berardi S Ria R Reale A De Luisi A Catacchio I Moschetta M. Multiple myeloma macrophages: pivotal players in the tumor microenvironment. J Oncol. (2013) 2013:183602. doi: 10.1155/2013/183602, PMID: 23431298 PMC3570938

[23] Cencini E Sicuranza A Ciofini S Fabbri A Bocchia M Gozzetti A. Tumor-associated macrophages in multiple myeloma: key role in disease biology and potential therapeutic implications. Curr Oncol. (2023) 30:6111–33. doi: 10.3390/curroncol30070455, PMID: 37504315 PMC10378698

[24] Sun J Park C Guenthner N Gurley S Zhang L Lubben B. Tumor-associated macrophages in multiple myeloma: advances in biology and therapy. J Immunother Cancer. (2022) 10(4):e003975. doi: 10.1136/jitc-2021-003975, PMID: 35428704 PMC9014078

[25] Kim J Denu RA Dollar BA Escalante LE Kuether JP Callander NS. Macrophages and mesenchymal stromal cells support survival and proliferation of multiple myeloma cells. Br J Haematol. (2012) 158:336–46. doi: 10.1111/j.1365-2141.2012.09154.x, PMID: 22583117 PMC3395762

[26] De Beule N De Veirman K Maes K De Bruyne E Menu E Breckpot K. Tumour-associated macrophage-mediated survival of myeloma cells through STAT3 activation. J Pathol. (2017) 241:534–46. doi: 10.1002/path.4860, PMID: 27976373

[27] Kovacs E. Interleukin-6 leads to interleukin-10 production in several human multiple myeloma cell lines. Does interleukin-10 enhance the proliferation of these cells? Leuk Res. (2010) 34:912–6. doi: 10.1016/j.leukres.2009.08.012, PMID: 19762082

[28] Alexandrakis MG Goulidaki N Pappa CA Boula A Psarakis F Neonakis I. Interleukin-10 induces both plasma cell proliferation and angiogenesis in multiple myeloma. Pathol Oncol Res. (2015) 21:929–34. doi: 10.1007/s12253-015-9921-z, PMID: 25743259

[29] Aras S Zaidi MR. TAMeless traitors: macrophages in cancer progression and metastasis. Br J Cancer. (2017) 117:1583–91. doi: 10.1038/bjc.2017.356, PMID: 29065107 PMC5729447

[30] Liu KX Joshi S. Re-educating” Tumor associated macrophages as a novel immunotherapy strategy for neuroblastoma. Front Immunol. (2020) 11:1947. doi: 10.3389/fimmu.2020.01947, PMID: 32983125 PMC7493646

[31] Beider K Bitner H Leiba M Gutwein O Koren-Michowitz M Ostrovsky O. Multiple myeloma cells recruit tumor-supportive macrophages through the CXCR4/CXCL12 axis and promote their polarization toward the M2 phenotype. Oncotarget. (2014) 5:11283–96. doi: 10.18632/oncotarget.2207, PMID: 25526031 PMC4294328

[32] Zavidij O Haradhvala NJ Mouhieddine TH Sklavenitis-Pistofidis R Cai S Reidy M. Single-cell RNA sequencing reveals compromised immune microenvironment in precursor stages of multiple myeloma. Nat Cancer. (2020) 1:493–506. doi: 10.1038/s43018-020-0053-3, PMID: 33409501 PMC7785110

[33] Smith LK Boukhaled GM Condotta SA Mazouz S Guthmiller JJ Vijay R. Interleukin-10 directly inhibits CD8(+) T cell function by enhancing N-glycan branching to decrease antigen sensitivity. Immunity. (2018) 48:299–312 e5. doi: 10.1016/j.immuni.2018.01.006, PMID: 29396160 PMC5935130

[34] Yan H Dong M Liu X Shen Q He D Huang X. Multiple myeloma cell-derived IL-32gamma increases the immunosuppressive function of macrophages by promoting indoleamine 2,3-dioxygenase (IDO) expression. Cancer Lett. (2019) 446:38–48. doi: 10.1016/j.canlet.2019.01.012, PMID: 30660652

[35] Dogra P Rancan C Ma W Toth M Senda T Carpenter DJ. Tissue determinants of human NK cell development, function, and residence. Cell. (2020) 180:749–63.e13. doi: 10.1016/j.cell.2020.01.022, PMID: 32059780 PMC7194029

[36] Stabile H Fionda C Gismondi A Santoni A. Role of distinct natural killer cell subsets in anticancer response. Front Immunol. (2017) 8:293. doi: 10.3389/fimmu.2017.00293, PMID: 28360915 PMC5352654

[37] Vivier E Tomasello E Baratin M Walzer T Ugolini S. Functions of natural killer cells. Nat Immunol. (2008) 9:503–10. doi: 10.1038/ni1582, PMID: 18425107

[38] Vulpis E Loconte L Peri A Molfetta R Caracciolo G Masuelli L. Impact on NK cell functions of acute versus chronic exposure to extracellular vesicle-associated MICA: Dual role in cancer immunosurveillance. J Extracell Vesicles. (2022) 11:e12176. doi: 10.1002/jev2.12176, PMID: 34973063 PMC8720178

[39] Seymour F Cavenagh JD Mathews J Gribben JG. NK cells CD56bright and CD56dim subset cytokine loss and exhaustion is associated with impaired survival in myeloma. Blood Adv. (2022) 6:5152–9. doi: 10.1182/bloodadvances.2022007905, PMID: 35834731 PMC9631618

[40] Sarkar S Germeraad WT Rouschop KM Steeghs EM van Gelder M Bos GM. Hypoxia induced impairment of NK cell cytotoxicity against multiple myeloma can be overcome by IL-2 activation of the NK cells. PLoS One. (2013) 8:e64835. doi: 10.1371/journal.pone.0064835, PMID: 23724099 PMC3665801

[41] Daly J Sarkar S Natoni A Stark JC Riley NM Bertozzi CR. Targeting hypersialylation in multiple myeloma represents a novel approach to enhance NK cell-mediated tumor responses. Blood Adv. (2022) 6:3352–66. doi: 10.1182/bloodadvances.2021006805, PMID: 35294519 PMC9198929

[42] Tomaipitinca L Russo E Bernardini G. NK cell surveillance of hematological Malignancies. Therapeutic implications and regulation by chemokine receptors. Mol Aspects Med. (2021) 80:100968. doi: 10.1016/j.mam.2021.100968, PMID: 34045078

[43] Cifaldi L Prencipe G Caiello I Bracaglia C Locatelli F De Benedetti F. Inhibition of natural killer cell cytotoxicity by interleukin-6: implications for the pathogenesis of macrophage activation syndrome. Arthritis Rheumatol. (2015) 67:3037–46. doi: 10.1002/art.39295, PMID: 26251193

[44] Lungu O Toscani D Giuliani N. Mechanistic insights into bone destruction in multiple myeloma: Cellular and molecular perspectives. J Bone Oncol. (2025) 51:100668. doi: 10.1016/j.jbo.2025.100668, PMID: 40124903 PMC11928850

[45] Mansour A Abou-Ezzi G Sitnicka E Jacobsen SE Wakkach A Blin-Wakkach C. Osteoclasts promote the formation of hematopoietic stem cell niches in the bone marrow. J Exp Med. (2012) 209:537–49. doi: 10.1084/jem.20110994, PMID: 22351931 PMC3302238

[46] Lawson MA McDonald MM Kovacic N Hua Khoo W Terry RL Down J. Osteoclasts control reactivation of dormant myeloma cells by remodelling the endosteal niche. Nat Commun. (2015) 6:8983. doi: 10.1038/ncomms9983, PMID: 26632274 PMC4686867

[47] Terpos E Zamagni E Lentzsch S Drake MT Garcia-Sanz R Abildgaard N. Treatment of multiple myeloma-related bone disease: recommendations from the Bone Working Group of the International Myeloma Working Group. Lancet Oncol. (2021) 22:e119–e30. doi: 10.1016/S1470-2045(20)30559-3, PMID: 33545067

[48] Tai YT Cho SF Anderson KC. Osteoclast immunosuppressive effects in multiple myeloma: role of programmed cell death ligand 1. Front Immunol. (2018) 9:1822. doi: 10.3389/fimmu.2018.01822, PMID: 30147691 PMC6095980

[49] An G Acharya C Feng X Wen K Zhong M Zhang L. Osteoclasts promote immune suppressive microenvironment in multiple myeloma: therapeutic implication. Blood. (2016) 128:1590–603. doi: 10.1182/blood-2016-03-707547, PMID: 27418644 PMC5034739

[50] Moreno L Perez C Zabaleta A Manrique I Alignani D Ajona D. The mechanism of action of the anti-CD38 monoclonal antibody isatuximab in multiple myeloma. Clin Cancer Res. (2019) 25:3176–87. doi: 10.1158/1078-0432.CCR-18-1597, PMID: 30692097

[51] Shah UA Mailankody S. Emerging immunotherapies in multiple myeloma. BMJ. (2020) 370:m3176. doi: 10.1136/bmj.m3176, PMID: 32958461

[52] Bisht K Fukao T Chiron M Richardson P Atanackovic D Chini E. Immunomodulatory properties of CD38 antibodies and their effect on anticancer efficacy in multiple myeloma. Cancer Med. (2023) 12:20332–52. doi: 10.1002/cam4.6619, PMID: 37840445 PMC10652336

[53] Martin TG Corzo K Chiron M Velde HV Abbadessa G Campana F. Therapeutic opportunities with pharmacological inhibition of CD38 with isatuximab. Cells. (2019) 8(12):1522. doi: 10.3390/cells8121522, PMID: 31779273 PMC6953105

[54] Kinder M Bahlis NJ Malavasi F De Goeij B Babich A Sendecki J. Comparison of CD38 antibodies *in vitro* and ex vivo mechanisms of action in multiple myeloma. Haematologica. (2021) 106:2004–8. doi: 10.3324/haematol.2020.268656, PMID: 33440920 PMC8252955

[55] Jiang H Acharya C An G Zhong M Feng X Wang L. SAR650984 directly induces multiple myeloma cell death via lysosomal-associated and apoptotic pathways, which is further enhanced by pomalidomide. Leukemia. (2016) 30:399–408. doi: 10.1038/leu.2015.240, PMID: 26338273

[56] Zhu C Song Z Wang A Srinivasan S Yang G Greco R. Isatuximab acts through fc-dependent, independent, and direct pathways to kill multiple myeloma cells. Front Immunol. (2020) 11:1771. doi: 10.3389/fimmu.2020.01771, PMID: 32922390 PMC7457083

[57] Barbieri E Maccaferri M Leonardi G Giacobbi F Corradini G Lagreca I. Adverse outcome associated with daratumumab-based treatments in relapsed/refractory multiple myeloma patients with amplification of chromosome arm 1q21: a single-center retrospective experience. Ann Hematol. (2022) 101:2777–9. doi: 10.1007/s00277-022-04978-6, PMID: 36104630 PMC9646578

[58] Martin T Richardson PG Facon T Moreau P Perrot A Spicka I. Primary outcomes by 1q21+ status for isatuximab-treated patients with relapsed/refractory multiple myeloma: subgroup analyses from ICARIA-MM and IKEMA. Haematologica. (2022) 107:2485–91. doi: 10.3324/haematol.2022.280660, PMID: 35734925 PMC9521209

[59] Nijhof IS Casneuf T van Velzen J van Kessel B Axel AE Syed K. CD38 expression and complement inhibitors affect response and resistance to daratumumab therapy in myeloma. Blood. (2016) 128:959–70. doi: 10.1182/blood-2016-03-703439, PMID: 27307294

[60] Saltarella I Desantis V Melaccio A Solimando AG Lamanuzzi A Ria R. Mechanisms of resistance to anti-CD38 daratumumab in multiple myeloma. Cells. (2020) 9(1):167. doi: 10.3390/cells9010167, PMID: 31936617 PMC7017193

[61] Ogiya D Liu J Ohguchi H Kurata K Samur MK Tai YT. The JAK-STAT pathway regulates CD38 on myeloma cells in the bone marrow microenvironment: therapeutic implications. Blood. (2020) 136:2334–45. doi: 10.1182/blood.2019004332, PMID: 32844992 PMC7702477

[62] Wu HT Zhao XY. Regulation of CD38 on multiple myeloma and NK cells by monoclonal antibodies. Int J Biol Sci. (2022) 18:1974–88. doi: 10.7150/ijbs.68148, PMID: 35342342 PMC8935232

[63] Franssen LE Stege CAM Zweegman S van de Donk N Nijhof IS. Resistance mechanisms towards CD38-directed antibody therapy in multiple myeloma. J Clin Med. (2020) 9(4):1195. doi: 10.3390/jcm9041195, PMID: 32331242 PMC7230744

[64] Ritchie D Colonna M. Mechanisms of action and clinical development of elotuzumab. Clin Transl Sci. (2018) 11:261–6. doi: 10.1111/cts.12532, PMID: 29272564 PMC5944582

[65] van de Donk N Usmani SZ. CD38 antibodies in multiple myeloma: mechanisms of action and modes of resistance. Front Immunol. (2018) 9:2134. doi: 10.3389/fimmu.2018.02134, PMID: 30294326 PMC6158369

[66] Wang Y Zhang Y Hughes T Zhang J Caligiuri MA Benson DM. Fratricide of NK cells in daratumumab therapy for multiple myeloma overcome by ex vivo-expanded autologous NK cells. Clin Cancer Res. (2018) 24:4006–17. doi: 10.1158/1078-0432.CCR-17-3117, PMID: 29666301 PMC6095810

[67] Casneuf T Xu XS Adams HC 3rd Axel AE Chiu C Khan I. Effects of daratumumab on natural killer cells and impact on clinical outcomes in relapsed or refractory multiple myeloma. Blood Adv. (2017) 1:2105–14. doi: 10.1182/bloodadvances.2017006866, PMID: 29296857 PMC5728278

[68] de Haart SJ Holthof L Noort WA Minnema MC Emmelot ME Aarts-Riemens T. Sepantronium bromide (YM155) improves daratumumab-mediated cellular lysis of multiple myeloma cells by abrogation of bone marrow stromal cell-induced resistance. Haematologica. (2016) 101:e339–42. doi: 10.3324/haematol.2015.139667, PMID: 27151995 PMC4967585

[69] Sun J Muz B Alhallak K Markovic M Gurley S Wang Z. Targeting CD47 as a novel immunotherapy for multiple myeloma. Cancers. (2020) 12(2):305. doi: 10.3390/cancers12020305, PMID: 32012878 PMC7072283

[70] van Bommel PE He Y Schepel I Hendriks M Wiersma VR van Ginkel RJ. CD20-selective inhibition of CD47-SIRPalpha “don’t eat me” signaling with a bispecific antibody-derivative enhances the anticancer activity of daratumumab, alemtuzumab and obinutuzumab. Oncoimmunology. (2018) 7:e1386361. doi: 10.1080/2162402X.2017.1386361, PMID: 29308308 PMC5749665

[71] Marlein CR Piddock RE Mistry JJ Zaitseva L Hellmich C Horton RH. CD38-driven mitochondrial trafficking promotes bioenergetic plasticity in multiple myeloma. Cancer Res. (2019) 79:2285–97. doi: 10.1158/0008-5472.CAN-18-0773, PMID: 30622116

[72] Fu Z Li S Han S Shi C Zhang Y. Antibody drug conjugate: the “biological missile” for targeted cancer therapy. Signal Transduct Target Ther. (2022) 7:93. doi: 10.1038/s41392-022-00947-7, PMID: 35318309 PMC8941077

[73] Tai YT Mayes PA Acharya C Zhong MY Cea M Cagnetta A. Novel anti-B-cell maturation antigen antibody-drug conjugate (GSK2857916) selectively induces killing of multiple myeloma. Blood. (2014) 123:3128–38. doi: 10.1182/blood-2013-10-535088, PMID: 24569262 PMC4023420

[74] Xing L Liu Y Liu J. Targeting BCMA in multiple myeloma: advances in antibody-drug conjugate therapy. Cancers. (2023) 15(8):2240. doi: 10.3390/cancers15082240, PMID: 37190168 PMC10137208

[75] Brudno JN Maric I Hartman SD Rose JJ Wang M Lam N. T cells genetically modified to express an anti-B-cell maturation antigen chimeric antigen receptor cause remissions of poor-prognosis relapsed multiple myeloma. J Clin Oncol. (2018) 36:2267–80. doi: 10.1200/JCO.2018.77.8084, PMID: 29812997 PMC6067798

[76] Sadelain M Brentjens R Riviere I. The basic principles of chimeric antigen receptor design. Cancer Discov. (2013) 3:388–98. doi: 10.1158/2159-8290.CD-12-0548, PMID: 23550147 PMC3667586

[77] Maher J Brentjens RJ Gunset G Riviere I Sadelain M. Human T-lymphocyte cytotoxicity and proliferation directed by a single chimeric TCRzeta/CD28 receptor. Nat Biotechnol. (2002) 20:70–5. doi: 10.1038/nbt0102-70, PMID: 11753365

[78] Sheykhhasan M Ahmadieh-Yazdi A Vicidomini R Poondla N Tanzadehpanah H Dirbaziyan A. CAR T therapies in multiple myeloma: unleashing the future. Cancer Gene Ther. (2024) 31:667–86. doi: 10.1038/s41417-024-00750-2, PMID: 38438559 PMC11101341

[79] Chekol Abebe E Yibeltal Shiferaw M Tadele Admasu F Asmamaw Dejenie T. Ciltacabtagene autoleucel: The second anti-BCMA CAR T-cell therapeutic armamentarium of relapsed or refractory multiple myeloma. Front Immunol. (2022) 13:991092. doi: 10.3389/fimmu.2022.991092, PMID: 36119032 PMC9479060

[80] Jagannath S Martin TG Lin Y Cohen AD Raje N Htut M. Long-term (≥5-year) remission and survival after treatment with ciltacabtagene autoleucel in CARTITUDE-1 patients with relapsed/refractory multiple myeloma. J Clin Oncol. (2025) doi: 10.1200/JCO-25-00760, PMID: 40459151 PMC12393059

[81] Rodriguez-Otero P Ailawadhi S Arnulf B Patel K Cavo M Nooka AK. Ide-cel or standard regimens in relapsed and refractory multiple myeloma. N Engl J Med. (2023) 388:1002–14. doi: 10.1056/NEJMoa2213614, PMID: 36762851

[82] Sidana S Patel KK Peres LC Bansal R Kocoglu MH Shune L. Safety and efficacy of standard-of-care ciltacabtagene autoleucel for relapsed/refractory multiple myeloma. Blood. (2025) 145:85–97. doi: 10.1182/blood.2024025945, PMID: 39365257 PMC11952008

[83] van de Donk N Themeli M Usmani SZ. Determinants of response and mechanisms of resistance of CAR T-cell therapy in multiple myeloma. Blood Cancer Discov. (2021) 2:302–18. doi: 10.1158/2643-3230.BCD-20-0227, PMID: 34386775 PMC8357299

[84] Leblay N. Cite-seq profiling of T cells in multiple myeloma patients undergoing BCMA targeting CAR-T or bites immunotherapy. Blood. (2020) 136(Supplement 1):11–2. doi: 10.1182/blood-2020-137650

[85] Holthof LC van der Schans JJ Katsarou A Poels R Gelderloos AT Drent E. Bone marrow mesenchymal stromal cells can render multiple myeloma cells resistant to cytotoxic machinery of CAR T cells through inhibition of apoptosis. Clin Cancer Res. (2021) 27:3793–803. doi: 10.1158/1078-0432.CCR-20-2188, PMID: 33883175

[86] Li W Zhang B Cao W Zhang W Li T Liu L. Identification of potential resistance mechanisms and therapeutic targets for the relapse of BCMA CAR-T therapy in relapsed/refractory multiple myeloma through single-cell sequencing. Exp Hematol Oncol. (2023) 12:44. doi: 10.1186/s40164-023-00402-5, PMID: 37158921 PMC10165782

[87] Zhang Q Bi J Zheng X Chen Y Wang H Wu W. Blockade of the checkpoint receptor TIGIT prevents NK cell exhaustion and elicits potent anti-tumor immunity. Nat Immunol. (2018) 19:723–32. doi: 10.1038/s41590-018-0132-0, PMID: 29915296

[88] Zeng Z Xu X Zhang Y Xing J Long J Gu L. Tumor-derived factors impaired motility and immune functions of dendritic cells through derangement of biophysical characteristics and reorganization of cytoskeleton. Cell Motil Cytoskeleton. (2007) 64:186–98. doi: 10.1002/cm.20175, PMID: 17183544

[89] Sakemura R Hefazi M Siegler EL Cox MJ Larson DP Hansen MJ. Targeting cancer-associated fibroblasts in the bone marrow prevents resistance to CART-cell therapy in multiple myeloma. Blood. (2022) 139:3708–21. doi: 10.1182/blood.2021012811, PMID: 35090171 PMC11290597

[90] Hu Y Hou J Jiang Z Lin Q. Mechanisms of resistance to CAR-T cell therapy in multiple myeloma: latest updates from the 2024 ASH annual meeting. Exp Hematol Oncol. (2025) 14:45. doi: 10.1186/s40164-025-00643-6, PMID: 40140947 PMC11938694

[91] Alabanza LM Xiong Y Vu B Webster B Wu D Hu P. Armored BCMA CAR T cells eliminate multiple myeloma and are resistant to the suppressive effects of TGF-beta. Front Immunol. (2022) 13:832645. doi: 10.3389/fimmu.2022.832645, PMID: 35222421 PMC8863610

[92] Hosny M Verkleij CPM van der Schans J Frerichs KA Mutis T Zweegman S. Current state of the art and prospects of T cell-redirecting bispecific antibodies in multiple myeloma. J Clin Med. (2021) 10(19):4593 . doi: 10.3390/jcm10194593, PMID: 34640611 PMC8509238

[93] Letouze E Moreau P Munshi N Samur M Minvielle S Touzeau C. Mechanisms of resistance to bispecific T-cell engagers in multiple myeloma and their clinical implications. Blood Adv. (2024) 8:2952–9. doi: 10.1182/bloodadvances.2023012354, PMID: 38513088 PMC11302375

[94] Truger MS Duell J Zhou X Heimeshoff L Ruckdeschel A John M. Single- and double-hit events in genes encoding immune targets before and after T cell-engaging antibody therapy in MM. Blood Adv. (2021) 5:3794–8. doi: 10.1182/bloodadvances.2021004418, PMID: 34471932 PMC8679680

[95] Lee H Ahn S Maity R Leblay N Ziccheddu B Truger M. Mechanisms of antigen escape from BCMA- or GPRC5D-targeted immunotherapies in multiple myeloma. Nat Med. (2023) 29:2295–306. doi: 10.1038/s41591-023-02491-5, PMID: 37653344 PMC10504087

[96] Verkleij CPM Broekmans MEC van Duin M Frerichs KA Kuiper R de Jonge AV. Preclinical activity and determinants of response of the GPRC5DxCD3 bispecific antibody talquetamab in multiple myeloma. Blood Adv. (2021) 5:2196–215. doi: 10.1182/bloodadvances.2020003805, PMID: 33890981 PMC8095149

[97] Meermeier EW Welsh SJ Sharik ME Du MT Garbitt VM Riggs DL. Tumor burden limits bispecific antibody efficacy through T cell exhaustion averted by concurrent cytotoxic therapy. Blood Cancer Discov. (2021) 2:354–69. doi: 10.1158/2643-3230.BCD-21-0038, PMID: 34258584 PMC8266040

[98] Friedrich MJ Neri P Kehl N Michel J Steiger S Kilian M. The pre-existing T cell landscape determines the response to bispecific T cell engagers in multiple myeloma patients. Cancer Cell. (2023) 41:711–25 e6. doi: 10.1016/j.ccell.2023.02.008, PMID: 36898378

[99] Verkleij CPM O’Neill CA Broekmans MEC Frerichs KA Bruins WSC Duetz C. T-cell characteristics impact response and resistance to T-cell-redirecting bispecific antibodies in multiple myeloma. Clin Cancer Res. (2024) 30:3006–22. doi: 10.1158/1078-0432.CCR-23-3333, PMID: 38687588

[100] Leblay N Maity R Hasan F Neri P. Deregulation of adaptive T cell immunity in multiple myeloma: insights into mechanisms and therapeutic opportunities. Front Oncol. (2020) 10:636. doi: 10.3389/fonc.2020.00636, PMID: 32432039 PMC7214816

[101] Ahn S Leblay N Neri P. Understanding the mechanisms of resistance to T cell-based immunotherapies to develop more favorable strategies in multiple myeloma. Hemasphere. (2021) 5:e575. doi: 10.1097/HS9.0000000000000575, PMID: 34095759 PMC8171358

[102] de Haart SJ van de Donk NW Minnema MC Huang JH Aarts-Riemens T Bovenschen N. Accessory cells of the microenvironment protect multiple myeloma from T-cell cytotoxicity through cell adhesion-mediated immune resistance. Clin Cancer Res. (2013) 19:5591–601. doi: 10.1158/1078-0432.CCR-12-3676, PMID: 24004671

[103] Chauhan D Singh AV Brahmandam M Carrasco R Bandi M Hideshima T. Functional interaction of plasmacytoid dendritic cells with multiple myeloma cells: a therapeutic target. Cancer Cell. (2009) 16:309–23. doi: 10.1016/j.ccr.2009.08.019, PMID: 19800576 PMC2762396

[104] Ramachandran IR Martner A Pisklakova A Condamine T Chase T Vogl T. Myeloid-derived suppressor cells regulate growth of multiple myeloma by inhibiting T cells in bone marrow. J Immunol. (2013) 190:3815–23. doi: 10.4049/jimmunol.1203373, PMID: 23460744 PMC3608837

[105] Gorgun GT Whitehill G Anderson JL Hideshima T Maguire C Laubach J. Tumor-promoting immune-suppressive myeloid-derived suppressor cells in the multiple myeloma microenvironment in humans. Blood. (2013) 121:2975–87. doi: 10.1182/blood-2012-08-448548, PMID: 23321256 PMC3624943

[106] Ray A Das DS Song Y Richardson P Munshi NC Chauhan D. Targeting PD1-PDL1 immune checkpoint in plasmacytoid dendritic cell interactions with T cells, natural killer cells and multiple myeloma cells. Leukemia. (2015) 29:1441–4. doi: 10.1038/leu.2015.11, PMID: 25634684 PMC5703039

[107] Horenstein AL Faini AC Morandi F Ortolan E Storti P Giuliani N. Monoclonal anti-CD38 therapy in human myeloma: retrospects and prospects. Front Immunol. (2025) 16:1519300. doi: 10.3389/fimmu.2025.1519300, PMID: 40013150 PMC11860881

[108] Storti P Vescovini R Costa F Marchica V Toscani D Dalla Palma B. CD14(+) CD16(+) monocytes are involved in daratumumab-mediated myeloma cells killing and in anti-CD47 therapeutic strategy. Br J Haematol. (2020) 190:430–6. doi: 10.1111/bjh.16548, PMID: 32162328

[109] Nijhof IS Groen RW Lokhorst HM van Kessel B Bloem AC van Velzen J. Upregulation of CD38 expression on multiple myeloma cells by all-trans retinoic acid improves the efficacy of daratumumab. Leukemia. (2015) 29:2039–49. doi: 10.1038/leu.2015.123, PMID: 25975191

[110] Perrot I Michaud HA Giraudon-Paoli M Augier S Docquier A Gros L. Blocking antibodies targeting the CD39/CD73 immunosuppressive pathway unleash immune responses in combination cancer therapies. Cell Rep. (2019) 27:2411–25.e9. doi: 10.1016/j.celrep.2019.04.091, PMID: 31116985

[111] Chemlal D Varlet E Machura A Ovejero S Requirand G Robert N. EZH2 targeting induces CD38 upregulation and response to anti-CD38 immunotherapies in multiple myeloma. Leukemia. (2023) 37:1925–8. doi: 10.1038/s41375-023-01983-0, PMID: 37532787 PMC10457196

[112] Liu J Xing L Li J Wen K Liu N Liu Y. Epigenetic regulation of CD38/CD48 by KDM6A mediates NK cell response in multiple myeloma. Nat Commun. (2024) 15:1367. doi: 10.1038/s41467-024-45561-z, PMID: 38355622 PMC10866908

[113] Ghermezi M Li M Vardanyan S Harutyunyan NM Gottlieb J Berenson A. Serum B-cell maturation antigen: a novel biomarker to predict outcomes for multiple myeloma patients. Haematologica. (2017) 102:785–95. doi: 10.3324/haematol.2016.150896, PMID: 28034989 PMC5395119

[114] Chen H Li M Xu N Ng N Sanchez E Soof CM. Serum B-cell maturation antigen (BCMA) reduces binding of anti-BCMA antibody to multiple myeloma cells. Leuk Res. (2019) 81:62–6. doi: 10.1016/j.leukres.2019.04.008, PMID: 31035033

[115] Swan D Madduri D Hocking J. CAR-T cell therapy in Multiple Myeloma: current status and future challenges. Blood Cancer J. (2024) 14:206. doi: 10.1038/s41408-024-01191-8, PMID: 39592597 PMC11599389

[116] Vieira dos Santos J Melnekoff D Aleman A Bhalla S Mouhieddine TH Van Oekelen O. Multimodal single-cell transcriptomic and proteomic correlatives of patients outcomes following anti-BCMA cellular therapy with ciltacabtagene autoleucel (Cilta-cel) in relapsed multiple myeloma. Blood. (2023) 142:93. doi: 10.1182/blood-2023-186395

[117] LeBlanc R Hideshima T Catley LP Shringarpure R Burger R Mitsiades N. Immunomodulatory drug costimulates T cells via the B7-CD28 pathway. Blood. (2004) 103:1787–90. doi: 10.1182/blood-2003-02-0361, PMID: 14512311

[118] Fostier K Caers J Meuleman N Broos K Corthals J Thielemans K. Impact of lenalidomide maintenance on the immune environment of multiple myeloma patients with low tumor burden after autologous stem cell transplantation. Oncotarget. (2018) 9:20476–89. doi: 10.18632/oncotarget.24944, PMID: 29755666 PMC5945510

[119] Lesokhin AM Tomasson MH Arnulf B Bahlis NJ Miles Prince H Niesvizky R. Elranatamab in relapsed or refractory multiple myeloma: phase 2 MagnetisMM-3 trial results. Nat Med. (2023) 29:2259–67. doi: 10.1038/s41591-023-02528-9, PMID: 37582952 PMC10504075

[120] Nooka AK Lesokhin AM Mohty M Niesvizky R Maisel C Arnulf B. Efficacy and safety of elranatamab in patients with relapsed/refractory multiple myeloma (RRMM) and prior B-cell maturation antigen (BCMA)-directed therapies: A pooled analysis from MagnetisMM studies. J Clin Oncol. (2023) 41:8008. doi: 10.1200/JCO.2023.41.16_suppl.8008

